# Enhancing the Flame Retardancy of Polyester/Cotton Blend Fabrics Using Biobased Urea–Phytate Salt

**DOI:** 10.3390/ma17061346

**Published:** 2024-03-14

**Authors:** Shuang Dong, Yi-Ting Huang, Xin Zhang, Shan-Shan Cheng, Xian-Wei Cheng, Jin-Ping Guan

**Affiliations:** 1 Key Laboratory of Flame Retardancy Finishing of Textile Materials (CNTAC), College of Textile and Clothing Engineering, Soochow University, 199 Renai Road, Suzhou 215123, China; ds314159265354@163.com (S.D.); 20194215029@stu.suda.edu.cn (Y.-T.H.);; 2 Zhongkang Guochuang Advanced Technology Research Institute of Dyeing and Finishing, Tai’an 271000, China; spc1155@126.com

**Keywords:** polyester/cotton, flame retardant, functional modification, phytate, biomass

## Abstract

The use of biobased flame-retardant (FR) agents for reducing the flammability of polyester/cotton (T/C) blend fabrics is highly desirable. In this study, a novel and sustainable phosphorus/nitrogen-containing FR, namely, phytic acid–urea (PA-UR) salt, was synthesized. The PA-UR salt was further used to enhance the FR performance of T/C fabric through surface modification. We further explored the potential chemical structure of PA-UR and the surface morphology, thermal stability, heat release capacity, FR properties, and mode of action of the coated fabric. The coated fabric achieved self-extinguishing and exhibited an increased limiting oxygen index of 31.8%. Moreover, the coated T/C blend fabric demonstrated a significantly reduced heat release capacity, indicating a decreased fire hazard. Thermogravimetric analysis revealed the anticipated decomposition of the coated T/C blend fabric and a subsequent increase in thermal stability. The burned char residues also maintained their fiber shape structures, suggesting the presence of condensed FR actions in the PA-UR-coated T/C blend fabric.

## 1. Introduction

Fire disasters pose a significant threat to daily life due to the widespread use of combustible polymers such as textiles, plastics, and woods. Textiles, in particular, are extensively used in clothing, interior decoration, and industrial packaging. Therefore, the development of high-efficiency flame-retardant (FR) agents for textiles is highly desirable [1,2]. Among synthetic and natural fibers, polyester and cotton are the most commonly used. Polyester/cotton (T/C) blend fabrics are widely utilized in bedding, interior decoration textiles, and military battle suits due to their combination of comfort, breathability, high elastic recovery, and wrinkle resistance [3,4,5].

However, T/C blend textiles possess a low flame retardancy because of the highly susceptible melting nature of polyester and cellulose fibers. During combustion, cotton decomposes during the initial stage, providing fuel, while polyester decomposes at higher temperatures, providing continuous fuel for burning. Moreover, melted polyester tends to adhere to charred cellulose fibers, providing additional energy and fuel. Consequently, T/C blend textiles exhibit severe burning behavior due to the “scaffolding effect” [6], resulting in a high fire risk that limits their application.

To address this issue, many functional modification approaches have been applied to enhance the FR performance of T/C blend fabrics, considering the characteristics of polyester and cotton textiles. Commercial FR systems, such as cyclic phosphonate-based FR compounds for polyester fabrics and Pyrovatex CP for cotton fabrics, can be used individually or in combination. Recently, the layer-by-layer (LBL) assembly technique has gained attention for constructing intumescent FR systems for T/C blend fabrics [7]. For instance, poly(allylam- inehydrochloride)/sodium hexametaphosphate [8], poly(diallydimethylammonium chloride)/poly(acrylic acid)/poly (diallydimethyl- ammonium chloride)/ammonium polyphosphate (APP) [9], APP/colloidal silica [10], APP/chitosan [11], and polyethyleneimine/oxidized sodium [12] systems have been employed to develop FR coatings on T/C blend fabrics through LBL assembly.

With increasingly strict environmental regulations, there has been growing interest in biobased FR compounds [13,14], such as proteins [15], deoxyribonucleic acid [16], aromatic tannins [17], chitosan, and lignin [18]. Among these materials, phytic acid (PA), derived from grains and beans, has gained widespread application in fabricating FR functional polymeric materials. Moreover, PA has a high phosphorus content and can effectively catalyze the formation of a physical barrier on material surfaces [19,20]. Additionally, PA is a suitable anionic candidate for LBL assembly technology because of its high efficiency in combination with cationic compounds and because it serves as an acid source for intumescent FR systems [21]. LBL counterparts such as chitosan/PA and zirconium phosphate nanosheets/PA/poly(hexamethylene guanidine hydrochloride) have been used to fabricate intumescent FR coatings for T/C blend fabrics [22,23]. However, there is a need for more feasible and effective FR approaches to enhance the FR performance of T/C blend fabrics using PA.

In this study, an effective P/N-containing FR (PA-UR) was designed and prepared utilizing PA and urea. The ability of PA molecules to combine different states of nitrogen increases the amount of nitrogen carried by the PA, thereby enhancing the synergistic P/N FR effect. We further applied PA-UR to construct an FR coating for T/C blend fabric. The chemical structure of PA-UR was characterized, and the thermal performance and heat generation performance of the coated T/C blend fabrics were also investigated. Additionally, the FR performance and potential mode of action were also explored.

## 2. Experimental

### 2.1. Materials

The polyester/cotton (T/C) blend fabric (65T/35C, 100 g/m^2^) was purchased from Jinzhou Dongli Textile Co., Ltd., Jinzhou, China. The phytic acid (70% aqueous solution) was provided by Shanghai Macklin Biochemical Co., Ltd., Shanghai, China. The urea was provided by Shanghai Biochemical Technology Co., Ltd., Shanghai, China. The dicyandiamide, ethanol and ammonia were provided by Chinasun Specialty Products Co., Ltd., Changshu, China.

### 2.2. Preparation of the PA-UR and Coated T/C Blend Fabric

**Figure 1:**
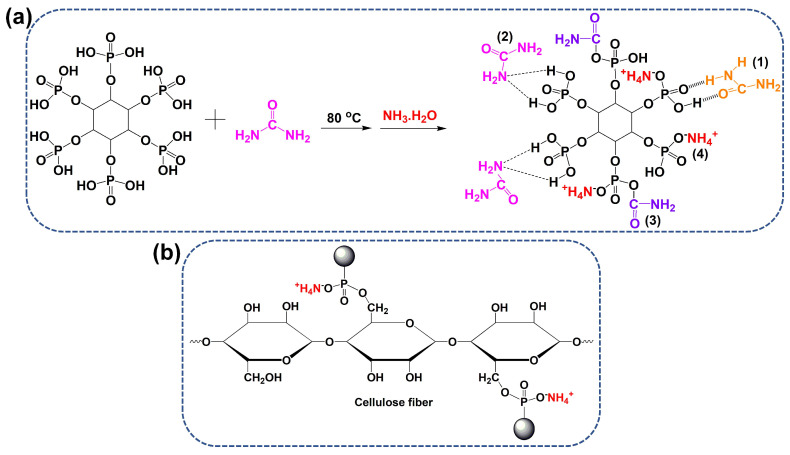
The potential reaction mechanism between PA and urea (**a**) and the potential cross-linking mechanism of PA-UR with cellulose fibers (**b**).

First, PA (0.01 mol, 9.4 g) and urea (0.06 mol, 3.6 g) were mixed together in a three-necked flask. The reaction was carried out at 80 °C for 4 h under magnetic stirring, affording a viscous, faint yellow product. The solvent was removed using a rotary evaporator. Finally, the crude product was rinsed several times with ethanol to obtain purified phytic acid–urea (PA-UR). The pure product was obtained at an 80% yield. The proposed synthetic route is displayed in Figure 1a.

PA-UR FR solutions at various concentrations (100~500 g/L) were prepared. Their pH was adjusted to a pH of 6 using ammonia. Dicyandiamide (50 g/L) was added to the solutions to catalyze the reaction between the ammonium phosphate groups and the hydroxyl groups of the cellulose fibers (Figure 1b) [24]. First, the T/C blend samples were immersed in FR solution and heated to 60 °C for 30 min, and they were squeezed through a lab-scale padder. The liquid-carrying capacity of the fabric reached 100 ± 5%. The squeezed samples were predried at 80 °C and baked at 160 °C for 3 min. Finally, the baked samples were washed and dried in air. The weight gain of the coated T/C blend fabric was determined in accordance with the weight of the coated and uncoated samples. The T/C blend samples coated with 150 and 300 g/L PA-UR were named T/C-1 and T/C-2, respectively.

### 2.3. Characterizations

The ^13^C and ^31^P liquid-state nuclear magnetic resonance (NMR) spectra of the FR agent were obtained using a Bruker Avance III 400 MHz spectrometer (Bruker BioSpin GmbH, Rheinstetten, Germany). The attenuated total reflection Fourier transform infrared (ATR/FT-IR) spectra of the samples were measured by means of a Nicolet iS50 FT-IR spectrometer (Thermo Fisher Scientific Inc., Waltham, MA, USA).

The surface morphologies of the T/C blend fabric and char residues were measured using a TM3030 tabletop scanning electron microscope at an accelerating voltage of 15 kV (Hitachi High Technologies America, Inc., Schaumburg, IL, USA), and a fitted energy disperse spectroscopy (EDS) spectrometer was applied for elemental analysis.

Thermogravimetry (TG) analysis of the T/C fabric was investigated using the TA Q600 SDT thermal analyzer (TA Instruments, New Castle, DE, USA) in both air and nitrogen atmospheres. Approximately 5 mg of powder was used, and the mixture was heated to 600 °C at a heating rate of 10 °C/min.

The heat release performance of the coated T/C fabric was evaluated via FTT0001 pyrolysis combustion flow calorimetry (PCFC) (Fire Testing Technology Ltd., East Grinstead, UK) according to ASTM D7309.

The limiting oxygen index (LOI) and vertical burning test were applied to investigate the flammability of the coated T/C samples. The LOI test was conducted using an FTT0080 oxygen index machine (Fire Testing Technology Ltd., East Grinstead, UK) with reference to GB/T 5454-1997 [25]. The vertical flammability was measured using a YG815B automatic vertical flammability cabinet (Ningbo Textile Instrument Factory, Ningbo, Zhejiang, China) with reference to the GB/T 5455-2014 standard [26]. The combustion grade was evaluated with reference to GB/T 17591-2006 [27].

The tensile strength of the T/C blend samples was measured using an Instron 3365 tester (Illinois Tool Works Inc., High Wycombe, Buckinghamshire, UK) with reference to ISO 13934-1-2013 [28]; the measurement was conducted 5 times to obtain an average value.

## 3. Results and Discussion

### 3.1. NMR and ATR/FT-IR of PA-UR

**Figure 2:**
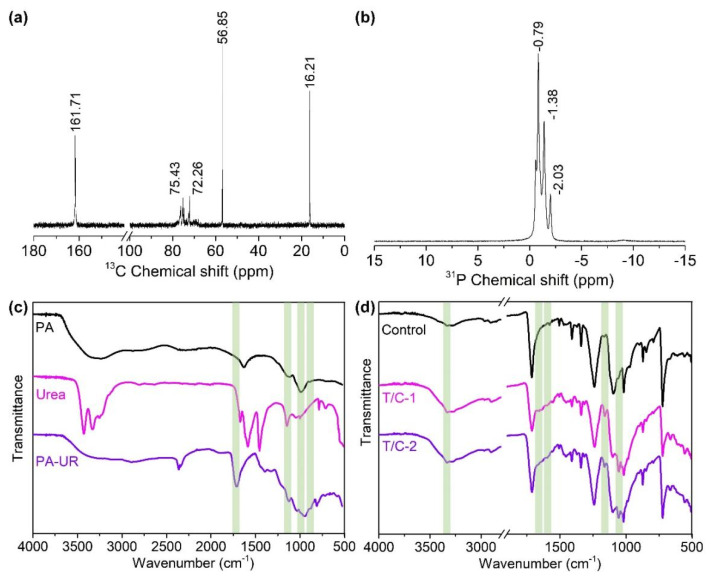
^13^C (**a**) and ^31^P (**b**) NMR spectra and ATR/FT-IR spectra of developed PA-UR (**c**) and coated T/C samples (**d**).

Figure 1a shows that four types of bonding that would occur between PA and urea, namely (1) hydrogen bonding, (2) ligand bonding, (3) chemical condensation, and (4) ionic bonding [29,30,31]. Figure 2a,b display the ^13^C and ^31^P NMR spectra of the synthesized PA-UR. The peaks at approximately 75.43 and 72.26 ppm in the ^13^C NMR spectrum should be assigned to the carbon atoms (C1~C6) of the inositol ring in PA. A weak ^13^C signal at 161.71 ppm is attributed to the residual urea carbonyl carbons through the hydrogen bonds with the phosphate roots in the PA-UR. The peaks at 56.85 and 16.21 ppm are assigned to the carbon atoms of the residual urea carbonyl carbons through the coordination bonds with the phosphate roots and O-C=O groups [29,30,31]. The ^31^P NMR spectrum shows P proton signals at −0.79, −1.38, and −2.03 ppm, indicating unreacted and reacted phosphate groups in different environments, which confirms the formation of new phosphate functional groups.

In the FT-IR spectrum of PA (Figure 2c), the stretching of the P=O and P-O groups occurs at approximately 1147 and 991 cm^−1^, respectively [32,33]. For the urea spectrum, the peaks at 3427 and 1672 cm^−1^ are ascribed to N-H stretching and deformation vibrations, respectively. The absorptions at 1587 and 1454 cm^−1^ can be attributed to C=O and C-N bonds, respectively [34]. However, the spectrum of PA-UR exhibited several different absorption peaks. Bands at 1147 and 944 cm^−1^ for the absorption of the P=O and P-O groups, respectively, are observed. The peak of the N-H groups shifts from 1672 cm^−1^ to 1708 cm^−1^ due to the changing environment. These absorption peaks are in good agreement with the structure of PA-UR (Figure 1a).

### 3.2. ATR/FT-IR and the Morphology of the Coated Fabric

As shown in Figure 2d, the changes in the absorption at approximately 3332 and 1672 cm^−1^ indicate the introduction of N-H groups by the FR coating. Furthermore, the spectra of the coated T/C blend samples exhibited new absorptions at approximately 1587, 1160, and 1050 cm^−1^, which correspond to the NH^4+^, C=O, P=O, and P-O structures in the PA-UR. This suggested the successful grafting of PA-UR onto the T/C blend fabric.

**Figure 3:**
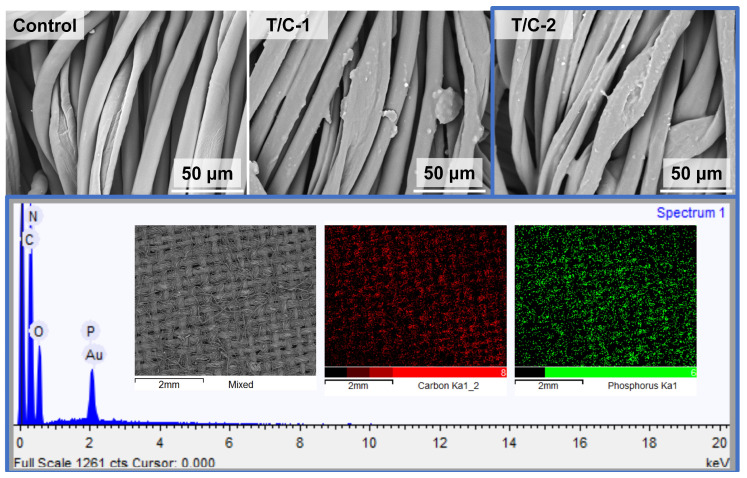
SEM micro-graphs and EDS maps of the coated T/C samples.

The surface morphology of the T/C blend fabric was evaluated using SEM (Figure 3). The uncoated fabric exhibited a clean surface, with regular smooth cylindrical fibers corresponding to the polyester fibers and fibers with slight cracks and dents representing the cellulose fibers. The coated T/C blend fibers displayed depositions and aggregations of FR compounds. The T/C-2 sample, with a weight gain of 17.9%, exhibited more robust depositions than did the T/C-1 sample, with a weight gain of 12.3%. The coated T/C blend samples also showed free gaps between the adjacent fibers. EDS mapping revealed that P was evenly distributed on the fibers, in addition to C and O. These results demonstrated the fine introduction of the FR coating onto the T/C fabric surface. The SEM images and EDS mapping supported the deposition of the PA-UR compound onto the T/C samples.

### 3.3. Thermal Performance

**Figure 4:**
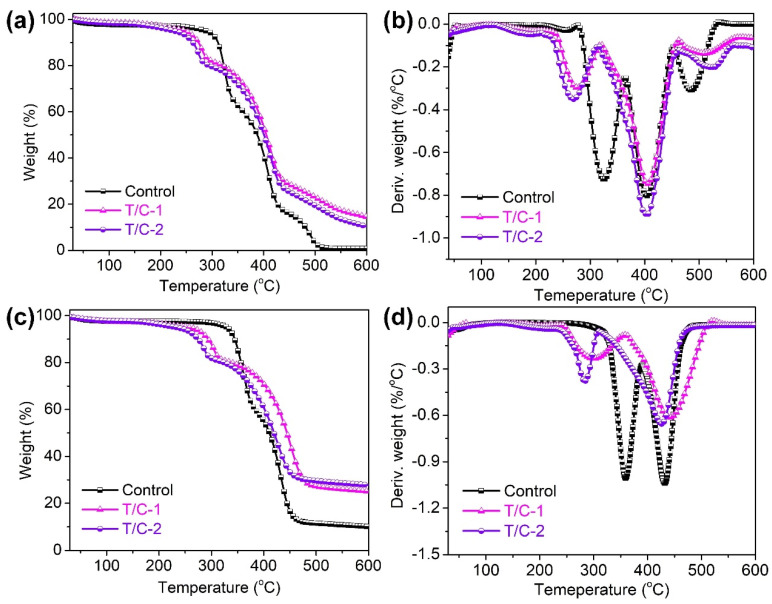
TG and DTG curves of the coated T/C samples under air (**a**,**b**) and nitrogen (**c**,**d**).

The thermal and thermal-oxidative stabilities of the coated T/C samples were estimated through TG analysis. Figure 4 displays the TG and derivative TG (DTG) curves, and Table 1 lists the key degradation data, including *T_5%_*, *T_max1_*, *T_max2_*, *T_max3_* (temperature at 5% and the first, second and third maximum weight loss), and the char residues at 600 °C. Three degradation stages were observed for the T/C blend fabric in air, while two degradation stages were observed in nitrogen. The first degradation stage at approximately 288.7 °C in air indicates the degradation of cotton fibers, while the second degradation stage at approximately 324.7 °C corresponds to the decomposition of polyester [9,35]. The coated T/C blend samples exhibited a thermal decomposition similar to that of the pristine fabric. However, the PA-UR coating accelerated the thermal degradation of the cotton portion, as suggested by the significantly lowered *T_5%_* and decreased *T_max1_* values listed in Table 1. The degradation pathway of the polyester fibers remained unchanged before and after the FR coating, as indicated by the unchanged *T_max2_* value.

It is hypothesized that the degradation of the FR agent in the early stage generated phosphoric acid and polyphosphoric acid, which acted as catalysts for the dehydration of the cellulose fibers, promoting the generation of a thermally stable char layer. This inhibited the exchange of heat, energy, and fuel between the gaseous and condensed phases, leading to the enhanced thermal stability and FR performance of the coated T/C samples. The third degradation stage in air can be attributed to the thermal-oxidative degradation of the carbohydrate polymers at higher temperatures. It was evident that the coated T/C blend samples exhibited a higher thermal stability in air, with increased *T_max3_* values and increased char residue at 600 °C. Furthermore, the thermal degradation of the coated T/C samples in nitrogen was similar to that in air, except for the absence of thermal-oxidative degradation.

**Figure 5:**
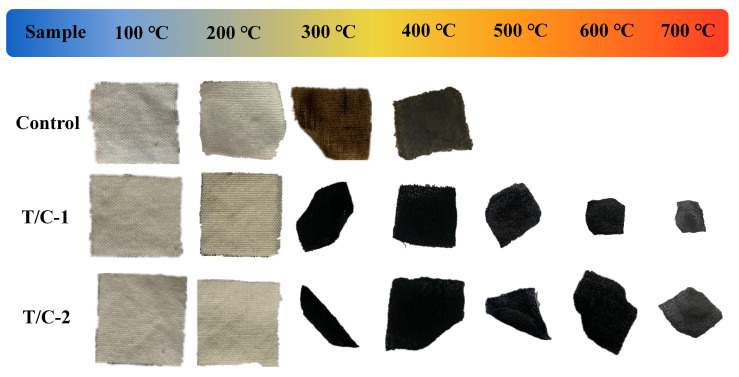
Digital images of coated T/C char residues after calcination in a muffle furnace.

Figure 5 clearly demonstrates that the coated T/C blend fabric exhibited earlier carbonization before 300 °C than did the pristine fabric. Moreover, it also displayed improved thermal stability at higher degradation temperatures, indicating that the FR properties of the PA-UR coating on the coated T/C blend fabric were in the condensed phase.

### 3.4. Heat Release Capacity

**Figure 6:**
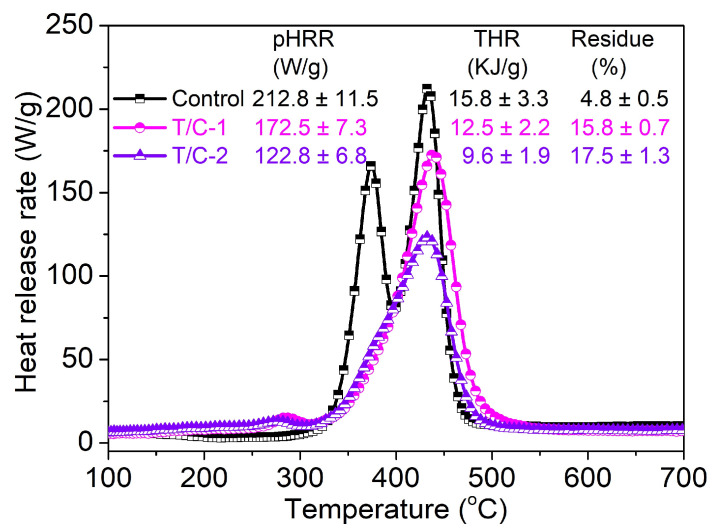
HRR curves and corresponding parameters of the coated T/C blend fabrics.

According to Figure 6, the pristine T/C fabric exhibited multiple peaks because of the presence of various organic components, which aligned well with the TG analysis. The first and second peaks of heat release were observed at 374.2 and 432.9 °C, with peak heat release rates (pHRR) of 166.1 and 212.8 W/g, respectively. Upon FR coating, the first heat release peak associated with cellulose degradation nearly disappeared. Additionally, the coated T/C blend fabric displayed a diminished second heat release peak, with pHRR reductions of 18.9% and 42.3% for T/C-1 and T/C-2, respectively. Similarly, the total heat release (THR) also showed a downward trend.

These findings demonstrated the effective suppression of heat release in the T/C blend fabric by the FR coating. This can be attributed to the shielding function of the thermal resistance protective layer, which hindered the exchange of energy and combustible species between the solid and gaseous phases. The increased residue amount observed in the coated T/C blend fabric indicated incomplete combustion, further contributing to the inhibition of heat release. Consequently, the coated T/C samples also displayed obviously decreased heat emissions.

### 3.5. Flame Retardancy

**Figure 7:**
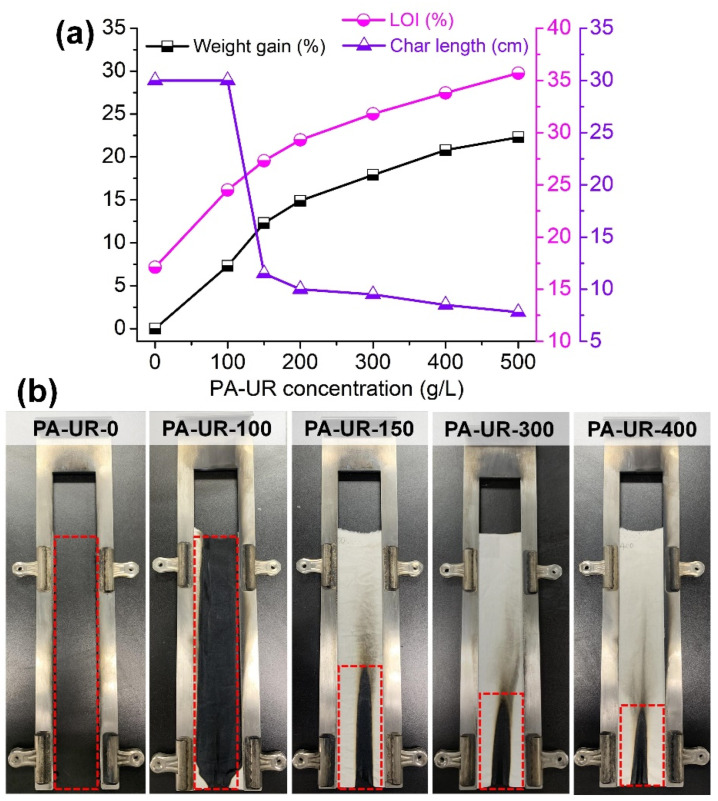
Weight gain, char length, and LOI of coated T/C samples (**a**) and images after vertical burning test (**b**).

To evaluate the FR performance of the coated T/C samples, vertical combustion and LOI tests were conducted. The corresponding results are presented in Figure 7. The pristine T/C blend fabric exhibited rapid combustion, with complete burnout occurring within 12 s of ignition. The occurrence of a “scaffolding effect” resulted in the formation of several melted residues at the boundaries of the device. The LOI of the pristine T/C blend fabric was low, measuring 17.1%, indicating a high fire hazard. Upon coating with 100 g/L PA-UR, the T/C blend fabric experienced a weight gain of 7.3% and retained a char residue with a textile structure (Figure 7b), despite complete combustion occurring.

The weight gain and FR performance of the coated T/C samples demonstrated an increasing trend with an increasing FR concentration (Figure 7a). The coated T/C blend fabric with PA-UR concentrations above 150 g/L exhibited self-extinguishing properties, with no after-flame or after-glow phenomena. Notably, the T/C-1 and T/C-2 samples, which experienced weight gains of 12.3% and 17.9%, respectively, achieved lower char lengths of 11.5 cm and 9.5 cm, meeting the B1 classification criteria. The T/C-1 and T/C-2 samples also exhibited high LOI values of 27.3% and 31.8%, respectively. However, the pure PA-treated T/C blend fabric burned completely, indicating the low efficiency of the FR effects on the T/C blend fabric. This confirmed that the coated T/C blend fabric possessed good flame retardancy, benefitting from the synergistic FR action of the phosphorous and nitrogen in the PA-UR [36].

**Figure 8:**
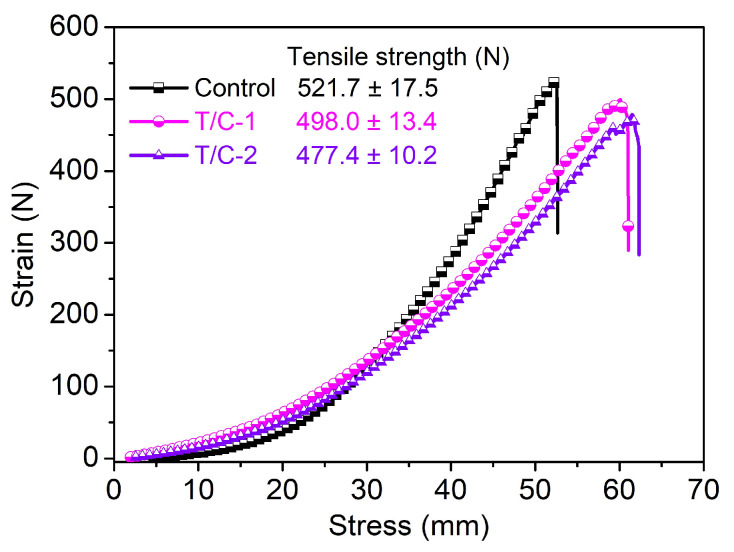
Stress-strain curves of the coated T/C samples.

Furthermore, it is important to note that the coated T/C sample T/C-2 exhibited complete combustion after 15 launderings, suggesting an unsatisfactory washing durability compared to that reported in previous studies. As reported, sufficient cross-linking of ammonium phosphate FR agents with cotton fibers adversely affects the tensile strength of cotton fabrics [24]. In comparison, the current coated T/C blend fabric had a lesser impacted tensile strength (Figure 8), likely because of the lower cellulose content and inadequate cross-linking between the PA-UR and T/C samples.

### 3.6. Char Residue Analyses

**Figure 9:**
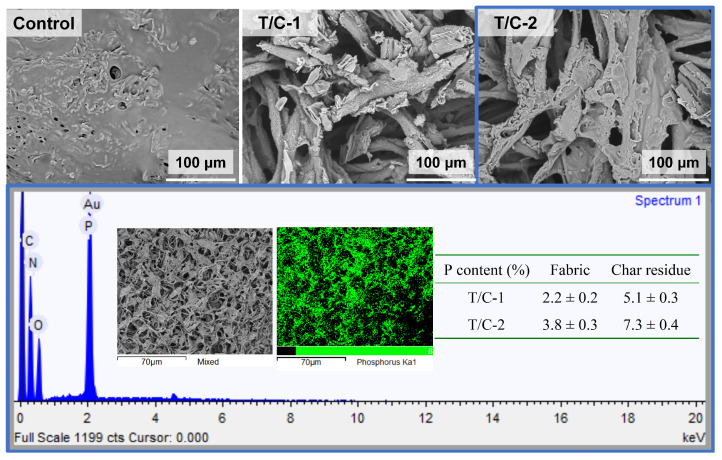
SEM micro-graphs and EDS maps of the coated T/C char residues.

As mentioned previously, the uncoated T/C sample exhibited intense burning due to the “scaffolding effect” of the polyester and cotton components. Consequently, the melted residues exhibited a matte surface with several holes (Figure 9), possibly arising from the nonuniform combustion of the cellulose and polyester. Interestingly, for the coated T/C blend samples, fiber shapes were observed on the burned samples, albeit with significant shrinkage. Additionally, the coated T/C blend samples exhibited intumescent char and inflated bubbles, which may serve as protective barriers to hinder the exchange of heat, fuel, and oxygen. This finding aligns with the self-extinguishing behavior observed in the coated T/C blend fabric.

Furthermore, EDS mapping revealed that P was finely dispersed on the char residues of the coated T/C blend fabric. Moreover, the T/C char residues exhibited an increased P content compared to the coated T/C samples. Specifically, the P contents of the T/C-1 and T/C-2 samples were 2.2% and 3.8%, respectively. However, the P contents of their corresponding char residues increased to 5.1% and 7.3%, respectively. This suggested that the P element primarily functioned during combustion and then remained in the condensed phase. The PA-UR coating effectively reduced the flammability of the T/C blend fabric through its condensed phase action.

## 4. Conclusions

This study developed an eco-friendly and effective FR coating, PA-UR, to enhance the FR performance of T/C blend fabrics. The chemical structure of the PA-UR was confirmed using ^13^C and ^31^P NMR and ATR/FT-IR analyses. Increasing the concentration of the FR coating resulted in greater weight gain and improved the FR ability of the coated T/C samples. The LOI values of the coated T/C samples exceeded 27.3%, and they exhibited a self-extinguishing ability at a weight gain of 12.3%. These results demonstrated the excellent FR efficiency of the PA-UR coating on the T/C blend fabric. Moreover, the FR coating significantly inhibited the heat release ability of the fabric. The coated T/C samples exhibited advanced thermal degradation at a lower temperature but greater thermal resistance at subsequent decomposition stages. This can be attributed to the early degradation of the PA-UR, which formed P-containing compounds that promoted the dehydration and carbonization of the T/C blend samples. The FR mechanism of PA-UR mainly involved a condensed FR mechanism, taking advantage of the synergistic effect of P and N. The FR coating displayed a slight influence on the tensile strength of the T/C blend fabric, indicating that PA-UR had a low degree of grafting onto a T/C blend fabric with low cellulose contents. Unfortunately, this also resulted in the poor laundering durability of the coated T/C samples. Further research is ongoing to enhance the laundering resistance of PA-based FR approaches to T/C blend fabrics to expand their potential application fields.

## Figures and Tables

**Table 1:** TG parameters of the coated T/C samples under air and nitrogen.

Samples		*T_5%_* (°C)	*T_max1_* (°C)	*T_max2_* (°C)	*T_max3_* (°C)	Residue at 600 °C (%)
Air	Control	288.7	324.7	404.0	485.2	0.8
	T/C-1	247.8	276.5	405.5	513.6	14.4
	T/C-2	226.5	267.6	402.6	524.8	10.3
Nitrogen	Control	329.5	369.8	434.7	—	10.1
	T/C-1	256.9	300.1	427.5	—	25.1
	T/C-2	232.5	283.1	441.6	—	27.8

## Data Availability

Data are contained within the article.
